# Study on Flake Formation Behavior and Its Influence Factors in Cr5 Steel

**DOI:** 10.3390/ma11050690

**Published:** 2018-04-27

**Authors:** Junkai Fan, Huitao Chen, Wu Zhao, Liang Yan

**Affiliations:** School of Mechanical and Power Engineering, Henan Polytechnic University, Jiaozuo 454000, China; junkaifan@hpu.edu.cn (J.F.); zhaowu@hpu.edu.cn (W.Z.); yanliang@hpu.edu.cn (L.Y.)

**Keywords:** flake, hydrogen embrittlement, trap hydrogen pressure, stress induced diffusion, Cr5VMo steel, finite element method

## Abstract

A flake is a crack that is induced by trapped hydrogen within steel. To study its formation mechanism, previous studies mostly focused on the formation process and magnitude of hydrogen pressure in hydrogen traps such as cavities and cracks. However, according to recent studies, the hydrogen leads to the decline of the mechanical properties of steel, which is known as hydrogen embrittlement, is another reason for flake formation. In addition, the phenomenon of stress induced hydrogen uphill diffusion should not be neglected. All of the three behaviors are at work simultaneously. In order to further explore the formation mechanism of flakes in steel, the process of flake initiation and growth were studied with the following three coupling factors: trap hydrogen pressure, hydrogen embrittlement, and stress induced hydrogen re-distribution. The analysis model was established using the finite element method, and a crack whose radius is 0.5 mm was set in its center. The cohesive method and Bilinear Traction Separate Law (BTSL) were used to address the coupling effect. The results show that trap hydrogen pressure is the main driving force for flake formation. After the high hydrogen pressure was generated around the trap, a stress field formed. In addition, the trap is the center of stress concentration. Then, hydrogen is concentrated in a distribution around this trap, and most of the steel mechanical properties are reduced. The trap size is a key factor for defining the critical hydrogen content for flake formation and propagation. However, when the trap size exceeds the specified value, the critical hydrogen content does not change any more. As for the crack whose radius is 0.5 mm, the critical hydrogen content of Cr5VMo steel is 2.2 ppm, which is much closer to the maximum safe hydrogen concentration of 2.0 ppm used in China. The work presented in this article increases our understanding of flake formation and propagation mechanisms in steel.

## 1. Introduction

**Figure 1:**
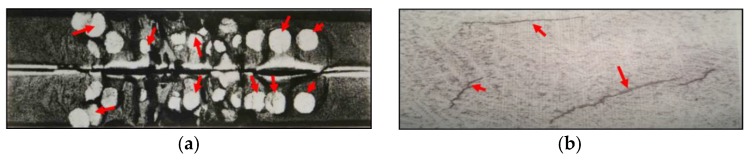
Flake shape in heavy forging: (**a**) flakes on a longitudinal section; (**b**) flakes on a cross section.

A flake is a crack generated in high strength steel with high hydrogen concentration, especially in heavy forging. Depending on their dimensions, number, and position in steel, flakes can drastically decrease the toughness and ductility of steel, and markedly reduce the service life of steel parts [1]. For heavy forging, once flakes are detected, the whole forging will be seen as a scraped product, which is a huge waste. The typical flake shape in heavy forging is shown in Figure 1.

Since flakes were discovered in steel, several hypotheses were proposed to explain their formation and propagation mechanism. Among them, the hydrogen pressure theory which was firstly proposed by Zapffe [2], and was recognized by most scholars. 

This theory holds that the high hydrogen pressure generated in traps is the reason for flake generation and growth. In steel, most of the hydrogen is dissolved in the lattice with the atom or ion state. However, there is still a considerable amount of hydrogen that is trapped in some inevitable flaws, which are known as hydrogen traps. Moreover, the trapped hydrogen atoms or ions can react to molecules within these traps, and apply an internal pressure on trap surfaces. As time progresses, a large amount of hydrogen diffuses into the trap’s form lattice, and the trap hydrogen pressure increases. This process can be continued for a long time until the chemical potential equilibrium is achieved between the molecular hydrogen in the trap and the atom or ion hydrogen in the lattice. Finally, a high hydrogen pressure is generated, and its magnitude is large enough to lead to flake formation and propagation. Recently, it has been proved that hydrogen is of the molecular form of the ion H_2_^+^ which can be dissolved in metals to form molecular H_2_ in traps [3,4].

After the hydrogen pressure theory was proposed, the focus of research is now how to define the pressure magnitude. Many studies have been done on this subject, and several hydrogen pressure calculation models have been developed [5,6,7,8,9,10]. However, among them, the hydrogen pressure calculation model, which was proposed by Kazinczy, is widely used because of its theoretical completeness and accuracy.

Flakes are very sensitive to hydrogen content, and they are only generated in steel with high hydrogen content. However, even if no flakes are formed, hydrogen is still very harmful to steel, especially to high strength steel. Many research studies show that hydrogen dissolved in lattice leads to drastically decreased material strength, especially in terms of the material fracture mechanical property. This phenomenon is called hydrogen embrittlement [11], and it can be discovered in many steels. 

In recent years, many researchers have focused their studies on the mechanisms of hydrogen embrittlement, and several hypotheses have been proposed, related to factors such as hydrogen-enhanced localized plasticity [12,13,14] and hydrogen-induced reductions in surface energy [15,16]. Among these mechanisms, hydrogen enhanced de-cohesion (HEDE) [17,18,19,20] is widely accepted in the literature. Based on this mechanism, a recent simulation model employing the finite element method was proposed and used by many researchers [21], especially and remarkably by Vigids Olden [22,23,24,25,26,27]. In addition, this simulation model is more effective and accurate than the analytical model [28,29,30,31,32,33]. 

The influence and mechanism of trapped hydrogen (hydrogen pressure in traps) and lattice hydrogen (hydrogen embrittlement) are well studied in steel, respectively. However, little work has been done to examine the two mechanisms working together for flake formation and propagation. The purpose of this paper is to study the flake formation and propagation mechanism with the three coupling factors: trap hydrogen pressure, hydrogen embrittlement, and stress induced hydrogen re-distribution. Furthermore, the influence factors on flake formation are also discussed. This work is processed based on the Cr5VMo steel and finite element method. We preliminarily focus on the problem of flake formation around an internal crack type flaw with a steady state condition. Further studies on flake formation in a transient condition will be summarized in our next study. These studies are very useful for understanding the interactions among flakes, hydrogen, and stress fields in steel.

## 2. Quantitative Model of the Three Mechanisms

### 2.1. Hydrogen Pressure in Trap

According to the hydrogen pressure theory, hydrogen in lattices can diffuse into traps and apply an internal pressure on trap surfaces. This process can continuously take place until a chemical potential equilibrium is achieved between lattice hydrogen and trapped hydrogen. Similarly, as the model developed by Kazinczy proposes, based on the chemical potential equilibrium relationship, the trap hydrogen pressure *p* can be deduced as [6]:

(1) $$c=MT\sqrt{p}\exp\left(-\frac{a-bp}{T}\right)$$

where *c* is the lattice hydrogen concentration measured in cm^3^ of hydrogen atoms per 100 g Fe, *p* is trap hydrogen pressure in dyne/cm^−2^, *T* is the temperature in K, *a* = 2300 K, *b* = 5.313 × 10^−7^ K/Pa, and *M* = 1.767 × 10^−5^ cm^4^/(100 g·K·dyne^1/2^).

### 2.2. Hydrogen Re-Distribution by Hydrostatic Stress

Stress can affect the hydrogen diffusion process, and this process is known as stress induced up-hill diffusion. Previous studies show that hydrostatic stress is the driving force of this diffusion phenomenon. Consequently, an extension of Fick’s law can be deduced as the governing equation for hydrogen diffusion, which accounts for the gradient of concentration and hydrostatic stress [23],

(2) $$\frac{\partial c}{\partial t}=D\nabla^{2}c+D\frac{V_{H}}{R(T-T^{Z})}\nabla c\nabla \sigma_{h}+D\frac{V_{H}}{R(T-T^{Z})}c\nabla^{2}\sigma_{h}$$

where *D* is the hydrogen diffusion coefficient, *V*_H_ = 2.0 × 10^3^ mm^3^/mol denotes the partial molar volume of hydrogen in iron-based alloys, *R* is the gas constant (8.314 J/mol·K), *T* is the actual temperature, *T*^Z^ is the absolute zero temperature, and *σ*_h_ is the hydrostatic stress. When this diffusion process has been achieved in steady state, the result of re-distributed lattice hydrogen can be expressed as:

(3) $$c_{h}=c\exp\left(\frac{\sigma_{h}V_{H}}{RT}\right)$$

### 2.3. Hydrogen Enhanced De-Cohesion (HEDE)

The HEDE mechanism is based on the hypothesis that interstitial hydrogen lowers the cohesive strength of the fracture energy by dilatation, which implies that hydrogen decreases the energy barrier for either grain boundary or cleavage plane de-cohesion. 

With the HEDE mechanism, material cohesive energy reduction is proportional to the local hydrogen concentration. Thus, the new variable, hydrogen coverage *θ* is introduced, which is a function of the actual hydrogen concentration and the Gibbs free energy difference between the interface and the surrounding material. The hydrogen coverage *θ* can be expressed in the Langmuir-McLean isotherm as [34]:

(4) $$\theta =\frac{c}{c+\exp\left(-\Delta g_{b}^{0}/RT\right)}$$

where $\Delta g_{b}^{0}$ is the Gibbs free energy. Serebrinsky [35] suggested a relation for the coupling between hydrogen coverage *θ* and the surface energy with hydrogen influence *γ*(*θ*), which can be expressed as:

(5) $$\gamma \left(\theta \right)=\left(1-1.0467\theta +0.1687\theta^{2}\right)\gamma \left(0\right)$$

where *γ*(0) is the energy necessary to create a new surface without hydrogen. Hence, assuming that the critical opening remains constant, the local critical hydrogen dependent cohesive stress can be given by:

(6) $$\frac{\sigma_{c}(\theta )}{\sigma_{c}(0)}=1-1.0467\theta +0.1687\theta^{2}$$

where *σ*_c_(0) and *σ*_c_(*θ*) are the local critical cohesive stress in the absence and presence of hydrogen. 

## 3. Finite Element Analysis Model

Hydrogen traps are widely existent in steel, and are of many shapes. However, the crack shape trap is most likely to develop into flakes. In this article, the simulation proceeds based on a crack model. The simulation model consists of the following three parts: elastic-plastic stress analysis, hydrogen diffusion analysis, and new crack generation analysis. The trap hydrogen pressure is applied on crack surfaces as a boundary condition, and its value is calculated based on Equation (1).

### 3.1. Traction Separation Law in Cohesive Method

Many previous studies show that the cohesive zone modeling (CZM) technique can be used in the finite element method to address the HEDE mechanism. This method assumes that fractures take place at an interface of cohesive zone elements, and new cracks can be generated on the interface when the judging criteria is achieved. The relevant constitutive of the material response is a traction separation description for an evaluation that gives the amount of energy required to create new surfaces. 

**Figure 2:**
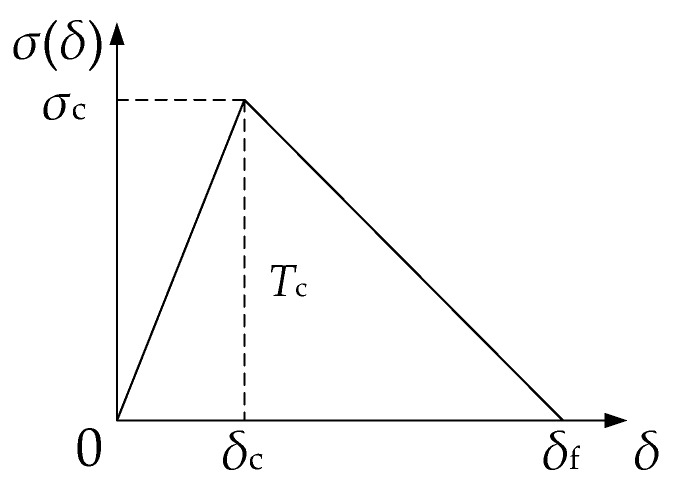
The bilinear cohesive zone model.

The kernel of CZM is the Traction Separation Law (TSL), which is a function described by the cohesive stress *σ*_c_ and separation *δ*. The area below the curve represents the cohesive energy *T*_c_. The Bilinear Traction Separate Law (BTSL), applied in the present paper, is shown in Figure 2. The formulation is:

(7) $$\sigma \left(\delta \right)=\left\{ \begin{array}{ll} \frac{\sigma_{c}}{\delta_{c}}\delta & \left(\delta \leq \delta_{c}\right)\\ \frac{\sigma_{c}(\delta_{f}-\delta )}{\delta_{f}-\delta_{c}} & \left(\delta >\delta_{c}\right)\\ 0 & \mathrm{otherwise} \end{array}\right.$$

The separation energy for the cohesive element can be described by:

(8) $$T_{c}=\frac{1}{2}\sigma_{c}\delta_{c}$$

In addition, the critical stress intensity factor can be defined by means of:

(9) $$K_{\mathrm{IC}}={\left(\frac{E\cdot T_{c}}{1-\upsilon^{2}}\right)}^{1/2}$$

### 3.2. Finite Element Model

**Figure 3:**
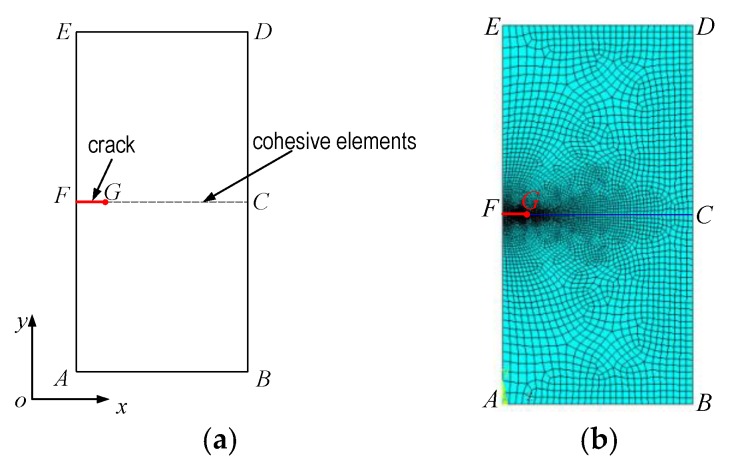
Geometry and FE mesh model: (**a**) geometry model; (**b**) mesh model.

The finite element analysis model is shown in Figure 3, which is a 2D axial symmetry model with a crack in its center. The radius of this crack is 0.5 mm, and the cohesive elements are placed on the crack plane. 

**Figure 4:**
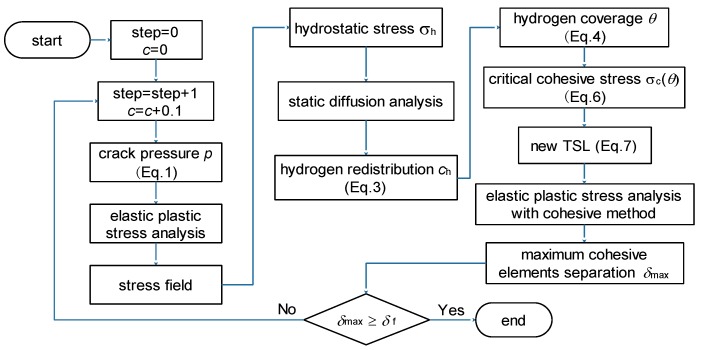
Simulation flow chat.

At the beginning of the simulation, the lattice hydrogen content was set as 0.1 ppm. After that, this value was updated with an increment of 0.1 ppm at each analysis step until new crack surfaces were generated. In each analysis step, the results of the stress field, hydrogen re-distribution, and cohesive strength were calculated. The simulation flow chart is shown in Figure 4.

The same mesh was applied in all three analysis steps. In the stress analysis, 4 nodes bilinear axial symmetry elements were used (Figure 3b). In the cohesive analysis, user-defined cohesive elements were added. Around the crack tip, the length of the cohesive element was 10–50 μm.

**Figure 5:**
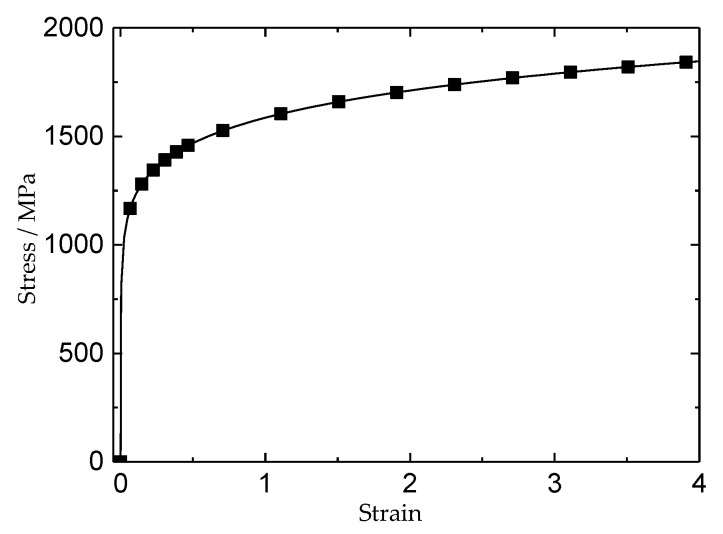
Strain-stress curve of Cr5VMo steel (calculated by JmatPro at 20 °C).

The Simulation proceeded based on Cr5VMo steel, which is widely used to produce roll forging in China, and is very sensitive to hydrogen. The chemical composition of Cr5VMo steel is shown in Table 1. All analyses proceeded at 20 °C, because 20 °C is the most likely temperature for flake formation and growth. The mechanical property of Cr5VMo steel at 20 °C was calculated by the JmatPro, which is a very effective calculation software of material properties (Figure 5). The steel yield stress was defined at 0.2% total strain, which is 1012 MPa, Young’s modulus was taken as 210 GPa, and Poisson’s ratio was 0.3.

To implement the BTSL law, some additional material properties should be determined. As for the cohesive stress *σ*_c_, a value of 4 times of material yield stress was used. The Gibbs free energy $\Delta g_{b}^{0}$ was set to 30 kJ/mol, which was set similarly by Serebrinsky [35]. Based on a fitting procedure to the experimental results, the separation value of *δ*_c_ = 2.0 × 10^−4^ mm was chosen for the BTSL law [23]. At 20 °C, the Cr5VMo steel critical stress intensity factor was 48.7 MPa·m^1/2^ [36], and the critical *δ*_f_ can be deduced from Equation (9) as:

(10) $$\delta_{f}=\frac{2K_{\mathrm{IC}}^{2}(1-\upsilon^{2})}{E\sigma_{c}}$$

## 4. Results

### 4.1. Influence of Hydrogen on Cohesive Model

**Figure 6:**
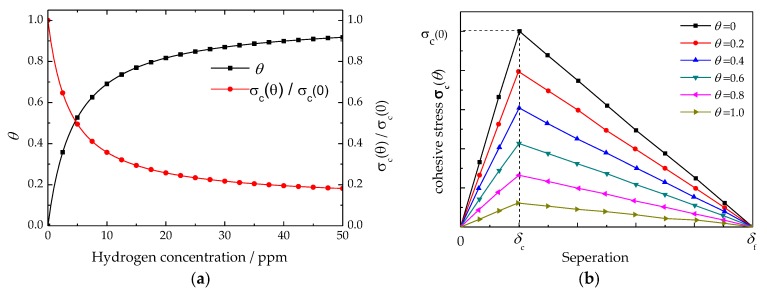
Variation of cohesive model parameters vs. hydrogen content: (**a**) hydrogen coverage and critical cohesive stress; (**b**) bilinear cohesive zone model.

The effect of hydrogen on the material cohesive strength can be reflected by the variation of critical cohesive stress. In Figure 6a, the influences of hydrogen content on hydrogen coverage and critical cohesive stress are shown graphically. The results indicate that hydrogen concentration has a great effect on critical cohesive stress, especially for the condition of hydrogen concentration below 20 ppm. With hydrogen concentration raising from 0 to 20 ppm, the hydrogen coverage was increased from 0 to 0.8, and the ratio of critical cohesive stress between the situations with and without hydrogen decreased from 1.0 to 0.23. As for the condition of hydrogen content above 20 ppm, the hydrogen coverage and critical cohesive stress were varied very slowly. Even hydrogen concentration improved from 20 ppm to 50 ppm, the hydrogen coverage was only reduced about 0.1, and the ratio of critical cohesive stress between situations with and without hydrogen was only reduced about 0.04. Figure 6b shows the influence of hydrogen coverage on the bilinear traction separation law. Here, the assumption was that hydrogen only affects the critical cohesive stress of the BTSL, and that critical and failure separations were constant. These results imply that hydrogen can affect critical stress and cohesive energy. With increasing hydrogen concentration, the hydrogen coverage improved. As a result, the critical stress and cohesive energy of the BTSL decreased.

### 4.2. Trap Hydrogen Pressure vs. Hydrogen Content

**Figure 7:**
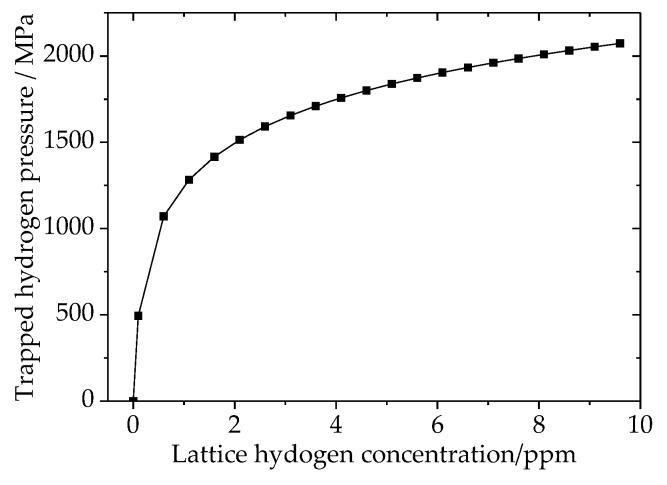
Trap hydrogen pressure variation vs. lattice hydrogen content.

The magnitude of trap hydrogen pressure is mostly dependent on lattice hydrogen concentration and temperature. When the temperature is 20 °C, the results of trap hydrogen pressure vary with lattice hydrogen content as shown in Figure 7. The results indicate that trap hydrogen pressure is very sensitive to lattice hydrogen content. With increasing lattice hydrogen content from 0 ppm to 2 ppm, the trap hydrogen pressure drastically improved from 0 to about 1500 MPa which greatly exceeds the limit of strength of many metals. 

### 4.3. Stress Induced Hydrogen Re-Distribution around Crack Tip

**Figure 8:**
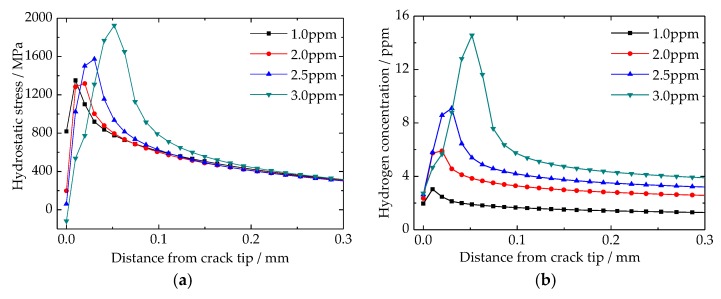
Distributions of hydrostatic stress and hydrogen content around crack tip: (**a**) hydrostatic stress; (**b**) hydrogen content.

With the increases of hydrogen content, the results of hydrostatic stress around the crack tip are shown in Figure 8a. It is interesting to find that the maximum hydrostatic stress is not located at crack tip, but rather a distance existed between the crack tip and maximum hydrostatic stress location. By improving the hydrogen content, this distance was enlarged and the crack tip hydrostatic stress decreased. According to the up-hill diffusion theory, the hydrostatic stress provides a new driving force for hydrogen diffusion. The results of hydrogen re-distribution around the crack tip are shown in Figure 8b. Similar to the results of hydrostatic stress, the maximum hydrogen content was located in front of the crack tip, not at the crack tip. In addition, the more hydrogen content, the longer the distance between the crack tip and the location of maximum hydrogen content. However, hydrogen re-distribution was more sensitive to hydrogen content than hydrostatic stress. It should be emphasized that hydrogen content at the crack tip was insensitive to any changes of average hydrogen concentration. The crack tip hydrogen content made small change around 2 ppm, and even the average hydrogen concentration increased 3 times from 1.0 ppm to 3.0 ppm.

### 4.4. Steel Cohesive Strength and Tensile Stress around Crack Tip

**Figure 9:**
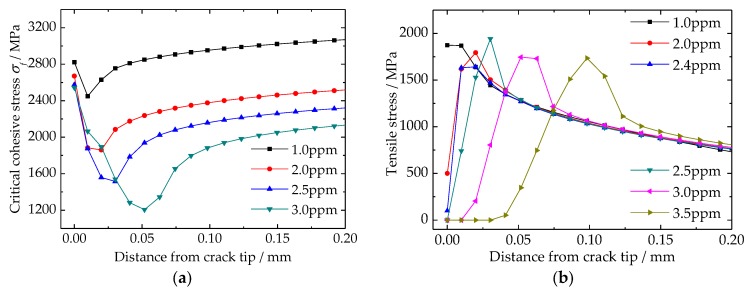
Distributions of critical cohesive stress and tensile stress around crack tip: (**a**) critical cohesive stress; (**b**) tensile stress.

According to the hydrogen enhanced de-cohesion mechanism (HEDE) and Bilinear Traction Separate Law (BTSL), the influence of hydrogen can be directly reflected by the variation of critical cohesive stress. Corresponding to the results of hydrogen distribution as shown in Figure 8b, the results of material critical cohesive stress are shown in Figure 9a. The location of critical cohesive stress’s biggest drop is the same as hydrogen distribution as shown in Figure 8b. In addition, it can be deduced that the critical cohesive stress is very sensitive to hydrogen content. As the average hydrogen content increased from 1 ppm to 3 ppm, the minimum critical cohesive stress is decreased from 2450 to 1200 MPa, respectively. However, this value at crack tip is reduced slowly, and it is only dropped from 2850 to 2550 MPa, accordingly. Figure 9b is the results of tensile stress around crack tip. The tensile stress is the result of high hydrogen pressure in crack, and it is the main reason for new crack surface generation or flake formation. The results show that location of the maximal tensile stress is getting further away from the crack tip, and its value is changing relative small, with increasing of the average hydrogen content. Tensile stress at crack tip is decreased when the average hydrogen content is increasing. In addition, the zone of none tensile stress at crack tip indicates that new crack surface is generated, in other word, the flake is formed. 

### 4.5. Critical Hydrogen Content of Flake Formation

**Figure 10:**
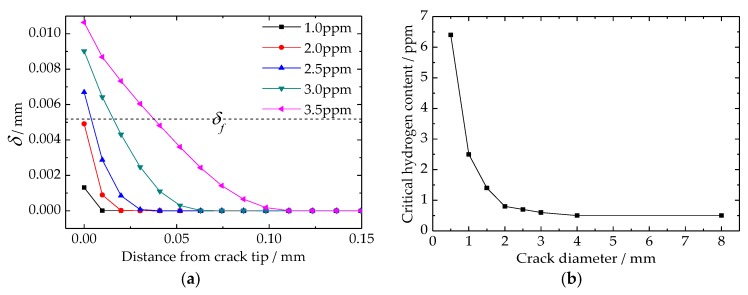
Critical hydrogen content for flake formation in Cr5VMo steel: (**a**) separation of the cohesive element around the crack tip; (**b**) influence of crack diameter.

The separation of cohesive elements around the crack tip can be used as the criteria of new crack surface generation. Figure 10a is the result of cohesive element separation, and the dotted line shown in this figure is the critical separation of flake formation. As for the results above the dotted line, the horizontal length of each line implies the length of a new generated flake. According to the results, it can be deduced that flakes are only generated at the crack tip, even though the maximum tensile stress and minimum critical cohesive stress are not all located at the crack tip. When the radius of the crack was 0.5 mm, the critical hydrogen content of flake formation was between 2.0 and 2.5 ppm. Besides the analysis model proposed by this article, several simulations are also run to study the relationship between the critical hydrogen content of flake formation and the crack diameter, and the results are shown in Figure 10b. The results indicate that crack size is a very important factor in the flake formation process. However, the influence of crack size can be divided into three parts. The first part is crack diameter below 2 mm, in which the critical hydrogen content increased drastically with decreasing crack size. When the crack diameter is 1 mm, the critical hydrogen content was about 2.2 ppm. However, when the crack diameter decreased to 0.5 mm, the critical hydrogen content increased drastically to about 6.5 ppm. As the crack diameter was between 2 mm and 4 mm, the critical hydrogen content reduced slowly. However, when the crack diameter was larger than 4 mm, the critical hydrogen content basically no longer changed, and its value was constant at 0.5 ppm. 

## 5. Discussion

It is generally accepted that hydrogen can lead to the degradation of steel mechanical property, and can be reflected in the reduction of steel fracture toughness. Fortunately, the varying of steel mechanical properties can be well addressed by the traction separation law. Through the variable of hydrogen coverage, the influence of hydrogen concentration can be well quantified (Figure 6a). In addition, hydrogen coverage can be used to define the BTSL critical cohesive stress (Figure 6b).

The cohesive method is widely used to simulate hydrogen embrittlement based on the hydrogen enhanced de-cohesion mechanism. However, little research takes the trap hydrogen pressure into account. Kazinczy’s investigation shows that hydrogen can be gathered in the flaws within steel, and applies a high hydrogen pressure on the flaws’ internal surface. For a steel part without any load, the trap hydrogen pressure is the driving force for new crack generation and crack propagation. In addition, this pressure magnitude is very sensitive to the surrounding lattice hydrogen concentration, and it can be increased to a very high value (Figure 7). The work proposed in this article is very different from previous studies, which in contrast take lattice hydrogen as the only reason for hydrogen induced delayed fractures in steel. 

The generated high hydrogen pressure in traps leads to a high stress field around the traps (Figure 8a and Figure 9b). Because of the up-hill diffusion of hydrogen, hydrogen is re-distributed around crack tips (Figure 8b). In addition, the material critical cohesive stress is reduced based on the results of the hydrogen distribution (Figure 9a). All three behaviors occur based on the generated high hydrogen pressure. It should be emphasized that the hydrogen aggregation around cracks is another reason for flake formation. The aggregated hydrogen can drastically decrease the material critical cohesive stress (Figure 9a). In addition, the form and degree of hydrogen aggregation largely depends on the distribution of the hydrostatic stress field, which is the result of the generated high hydrogen pressure in cracks (Figure 9b). 

Although the maximum hydrogen content and tensile stress are not located at crack tip, the new crack surface is only generated at the crack tip (Figure 10a). Trap size is a key factor for flake formation. The longer of crack length, the less hydrogen concentration is needed to generate a new crack surface. However, this tendency only exists when the crack diameter is below 4 mm, and changes drastically within the interval from 0 to 2 mm (Figure 10b). As the crack diameter is above 4 mm, only 0.5 ppm hydrogen concentration is needed for flake formation. In addition, the new generated flake can continuously propagate without any more hydrogen. These results indicate that reducing and controlling the flaw size within steel are very effective ways to avoid flake generation. On the other hand, the ultrasonic testing is very important for many steel parts. In addition, the accuracy of the ultrasonic testing and hydrogen content can be used to predict the possibility of flake formation. It should be noted that the analysis model proposed in this article can only be used on the macroscopic scale, generally on the millimeter scale. Establishing a microscopic scale analysis model, especially based on the grain size, and studying the microscopic mechanism of flake initiation is the focus of our next research project.

## 6. Conclusions

In this article, the three factors of trap hydrogen pressure, stress induced hydrogen re-distribution, and hydrogen embrittlement are firstly coupled to study the mechanism of flake formation and propagation in Cr5VMo steel using the finite element method. From our results, three main conclusions can be drawn. The first is that trap hydrogen pressure is the main driving force for flake formation in steel. It not only defines the stress field around crack tips, but also affects the degree of hydrogen aggregation in steel. The second is that trap size is a very important factor for defining the critical hydrogen content of flake formation. The ultrasonic testing and its accuracy are very important for predicting the possibility of flake formation. The third conclusions is that hydrogen content is the key to flake initiation, and its value should be well examined and controlled. In addition, hydrogen detection accuracy should be guaranteed effectively, especially as the hydrogen content is below 3 ppm. The simulation method and results proposed in this article generally support the understanding of hydrogen assisted cracking phenomena in steel. They are helpful for developing appropriate measures for preventing flake formation in the future.

## Figures and Tables

**Table 1:** The chemical composition of Cr5VMo steel.

Element	Fe	Cr	Mn	Mo	Ni	Si	V	C	P
wt %	92.94	4.5	0.5	0.5	0.4	0.5	0.15	0.5	0.01
